# Anxiety and depression in patients wearing prosthetic eyes

**DOI:** 10.1007/s00417-020-04908-0

**Published:** 2020-09-01

**Authors:** Ludwig M. Heindl, Marc Trester, Yongwei Guo, Florian Zwiener, Narges Sadat, Nicola S. Pine, Keith R. Pine, Andreas Traweger, Alexander C. Rokohl

**Affiliations:** 1 Department of Ophthalmology, Faculty of Medicine, University Hospital of Cologne, University of Cologne, Kerpener Strasse 62, 50924 Cologne, Germany; 2 Center for Integrated Oncology (CIO) Aachen-Bonn-Cologne-Dusseldorf, Cologne, Germany; 3 Trester-Institute for Ocular Prosthetics and Artificial Eyes, Cologne, Germany; 4 Auckland District Health Board, Auckland, New Zealand; 5 School of Optometry and Vision Science, University of Auckland, Auckland, New Zealand; 6 Institute for Tendon and Bone Regeneration, Spinal Cord Injury and Tissue Regeneration Centre Salzburg, Paracelsus Medical University, Salzburg, Austria; 7 Austrian Cluster for Tissue Regeneration, Vienna, Austria

**Keywords:** Anxiety, Depression, Anophthalmia, Ocular prostheses, Cryolite glass prosthetic eyes, Integrated care

## Abstract

**Purpose:**

To investigate anxiety and depression levels in prosthetic eye–wearing patients using standardized psychometric instruments, to define factors associated with these psychological diseases, and to identify a potential healthcare gap.

**Methods:**

A total of 295 prosthetic eye wearers were screened using the 7-item generalized anxiety disorder scale (GAD-7) and the 9-item patient health questionnaire (PHQ-9). Scores of GAD-7 and PHQ-9 were correlated with scores of general physical and mental health functioning, vision-related quality of life, appearance-related distress, appearance-related social function, and further biosocial factors.

**Results:**

Five patients (2%) had a pre-diagnosed anxiety disorder, and 20 patients (7%) had a pre-diagnosed depression. However, our screening revealed 26 patients (9%) with anxiety symptoms, 31 patients (11%) with depression symptoms, and 40 patients (14%) suffering from both anxiety and depression symptoms. This underdiagnosing for both anxiety and depression disorders was significant (*p* < 0.001, respectively). Higher GAD-7 scores were significantly associated with higher PHQ-9 scores, lower appearance-related social function, lower mental health functioning, and female gender (*p* ≤ 0.021, respectively). Higher PHQ-9 scores were significantly associated with lower physical and mental health functioning, higher educational degree, and non-traumatic eye loss (*p* ≤ 0.038, respectively).

**Conclusions:**

Anxiety and depression disorders seem to be underdiagnosed in prosthetic eye wearers and to have higher incidence compared with the general population. Therefore, a psychometric screening should be routinely implemented in the clinical care. For a successful long-term rehabilitation, integrated care by a multidisciplinary team including ophthalmic plastic surgeons, ophthalmologists, ocularists, general practitioners, and psychologists is essential.

## Introduction


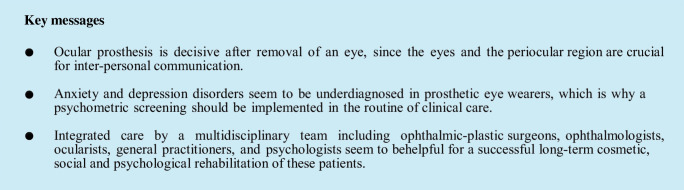
The eyes and the periocular region are crucial for inter-personal communication [1]. Although prosthetic eye wearers mostly express high levels of satisfaction with their eye prosthesis, living with an ocular prosthesis has a significant impact on psychosocial factors and social interactions [1–5].

Most psychological studies regarding prosthetic eye wearers focused on concerns of anophthalmic patients or quality of life–affecting issues in this ordinary population [1–20]. The eye loss itself, a potential malignant eye disease, the health of the fellow eye, potential discharge at the anophthalmic socket, dry socket symptoms, altered visual perception, and changes of the appearance have been already reported as relevant life-affecting and distress-causing factors [1–7, 13–18, 21–23].

Distressed patients typically exhibited higher levels of anxiety, depression, self-consciousness, and social avoidance especially within the first months after eye loss [24].

General anxiety disorder is one of the most common mental disorders with a lifetime prevalence of 2.8–7.3% in the general population, but often undetected by physicians [25–29]. Furthermore, general anxiety disorders have a significant comorbidity with depression, which shows a prevalence of 7.7% in the general German population [28, 30, 31].

Several community-based studies have already analyzed the prevalence of depression and anxiety in patients with various eye diseases and the influence of these diseases on daily life [29, 32, 33]. However, until today, there is no extensive and systematic study investigating general anxiety disorders and depression dependent on main influence factors including general health status, appearance-related distress, appearance-related social function, and vision-related quality of life in prosthetic eye wearers using standardized and established psychometric tools. In addition, there is no study addressing anxiety and depression in cryolite glass prosthetic eye wearers at all.

Therefore, the purpose of the present study is to evaluate anxiety and depression symptoms in patients wearing prosthetic eyes using standardized psychometric screening instruments, to define factors associated with these psychological diseases, and to identify a potential healthcare gap.

## Patients and methods

Over 63 consecutive working days between June 2019 and November 2019, patients who underwent ocular prosthetic care at the Trester-Institute for Ocular Prosthetics and Artificial Eyes, Cologne, Germany, were asked directly prior to their treatment to participate in an extensive study regarding anxiety and depression symptoms. The study was conducted by the Department of Ophthalmology, University of Cologne, Cologne, Germany, in adherence to the tenets of the Declaration of Helsinki and approved by the Institutional Review Board of the University of Cologne. In addition, this study was performed independently from previous studies. Informed consent was obtained from the subjects after explanation of the nature and possible consequences of the study. Inclusion criteria were age over 18, wearing cryolite glass prosthetic eyes, and adequate command of the German language.

Patients were asked face-to-face questions using a standardized three-section questionnaire: Section 1 requested general demographic data and information about age, gender, ethnicity, current relationship status, if the patient lives alone or not, highest educational degree, occupation, occupational disability, and amount of income.

In section 2, patients were asked about their history and treatment of already diagnosed active depression and anxiety-related disorders. Furthermore, a detailed ocular anamnesis for the anophthalmic site including time since the eye was lost, reason for eye loss, performed surgery, and age at time of eye loss was asked.

Section 3 included German versions of six standardized and established questionnaires for the psychometric evaluation of depression, anxiety, health-related quality of life, vision-related quality of life, appearance-related psychological distress, and appearance-related social interactions.

The patient health questionnaire (PHQ) is a self-administered version of the PRIME-MD diagnostic instrument for common mental disorders [34]. The PHQ-9 is a reliable, valid, and short depression screening and grading module, which scores each of the 9 DSM (Diagnostic and Statistical Manual of Mental Disorders) criteria as “0” (not at all) to “3” (nearly every day) [35]. PHQ-9 scores of 5, 10, 15, and 20 represent mild, moderate, moderately severe, and severe depression, respectively [35]. While the PHQ-9 scores and grades depression symptoms, it does not reveal reasons or type of the depression such as endogenous depression, depressive personality disorder, or exogenous (reactive) depression.

The 7-item generalized anxiety disorder (GAD-7) scale is a validated, reliable, and efficient measurement tool for GAD screening and assessing its severity in clinical practice and in research [34]. The GAD-7 scale classifies 4 levels of anxiety severity: none (0–4), mild (5–9), moderate (10–14), and severe (15–21) [34].

The 12-item short-form health survey (SF-12) is a shortened version of the 36-item short-form health survey [36]. SF-12 is a measurement tool of general physical and general mental health functioning that has been widely used and validated [36]. Responses to SF-12 questions were used to estimate a general mental composite score (MCS) and general physical composite score (PCS) for each patient, with higher values indicating higher health-related quality of life [36].

The 14-item visual function questionnaire (VF-14), one of the most commonly used vision-related functional questionnaires, is based on 14 vision-dependent activities performed in everyday life, and the difficulty undertaking each activity is rated [37, 38]. It has also been validated for use with a wide range of eye diseases [37, 38]. Scores range from 0 to 100, and higher scores represent better visual functioning and less difficulty to perform daily activities [37, 38].

The FACE-Q module is a patient-reported outcome instrument designed to measure important concepts of interest including various scales such as the appearance-related distress scale and appearance-related social function scale, evaluating social interactions [39, 40]. Both the appearance-related psychosocial distress scale and the appearance-related social function scale contain eight statements, respectively [39, 40]. Responses to each statement were rated on a four-point Likert-type scale and transformed to a score between 0 and 100 for each scale [39, 40]. Higher values represent a greater severity of psychosocial distress and a better social function [39, 40].

If the patients had any issues or understanding problems regarding the questions, these issues were clarified directly.

### Statistical analyses

A commercial software (SPSS version 26.0 for Mac; SPSS, Inc., Chicago, IL) was used for all statistical analyses. Kolmogorov–Smirnov tests were performed to analyze normal distribution of all scores. Due to not normal distribution, Mann–Whitney *U* tests were performed to compare the PHQ-9 and GAD-7 scores between symptomatic and non-symptomatic patients. To evaluate differences between the rates of previously diagnosed disease and of symptomatic patients, Wilcoxon tests were used after a grading of the patients as pre-diagnosed (or not) and as symptomatic (or not) was performed. To investigate factors related to the PHQ-9 and GAD-7 scores, general linear models were used (one for each questionnaire) with explanatory variables of physical (SF-12 PCS) and general mental health functioning (SF-12 MCS), appearance-related distress (FACE-Q appearance-related psychosocial distress scale), appearance-related social function (FACE-Q appearance-related social function scale), vision-related quality of life (VF-14 score), gender (male vs. female), age, age at eye loss, highest educational degree, ethnicity (European or not), occupational disability, reason for eye loss, relationship status (single or not), time since eye loss under 1 year (or not), and if patient lived alone (or not).

Both linear regression models were highly significant (ANOVA: *p* ≤ 0.001), and all *p* values of the regression coefficients < 0.05 were reported as statistically significant.

## Results

### Biosocial profile of 295 prosthetic eye wearers

Out of 324 patients who were approached to participate, 295 patients agreed; 29 patients declined to participate due to lack of time. Of these 295 patients who agreed, 192 were males and 103 were females (Table 1). These 295 enrolled patients had a mean age of 62.54 ± 16.77 years (range, 18–95 years). In total, 90.5% were European, 7.1% were from the Middle East, 1 patient was Latin-American, 3 were Asian, and 3 were African. Over 60% of the patients were married or in a relationship, while 38.3% were single, divorced, or widowed. In total, 92 (31%) patients lived alone. Nineteen patients (6.4%) had no educational degree, while the highest educational degree of 89 patients (30%) was secondary school or high school. A total of 125 patients (42%) had an apprenticeship and 51 patients (17%) a university degree. Most of the patients were retired (49.3%) or full-time employed (32.2%). Seventy-four percent made no statement regarding their amount of income.

**Table 1:** Biosocial profile of 295 unilateral anophthalmic patients wearing cryolite glass prosthetic eyes

Age, mean ± SD (range)	62.54 ± 16.77 (range, 18–95)
Gender	
Male, *n* (%)	192 (65.1%)
Female, *n* (%)	103 (34.9%)
Ethnicity	
European, *n* (%)	267 (90.5%)
Middle East, *n* (%)	21 (7.1%)
Asian, *n* (%)	3 (1.0%)
African, *n* (%)	3 (1.0%)
Latin-American, *n* (%)	1 (0.3%)
Current relationship status	
Married or in a relationship, *n* (%)	182 (61.7%)
Single, *n* (%)	113 (38.3%)
Living alone	
Yes, *n* (%)	92 (31.2%)
No, *n* (%)	203 (68.8%)
Highest educational degree	
No degree, *n* (%)	19 (6.4%)
Secondary school, *n* (%)	89 (30.2%)
High school, *n* (%)	11 (3.7%)
Apprenticeship, *n* (%)	125 (42.4%)
University degree, *n* (%)	51 (17.3%)
Job	
Self-employed, *n* (%)	12 (4.1%)
Employed full-time, *n* (%)	95 (32.2%)
Employed part-time, *n* (%)	15 (5.1%)
Retired, *n* (%)	148 (49.3%)
In training, *n* (%)	5 (3.3%)
No job, *n* (%)	20 (6.6%)
Occupational disability due to eye loss	
Yes, *n* (%)	16 (5.4%)
No, *n* (%)	279 (94.1%)
Income per month (Euros)	
< 1000, *n* (%)	18 (6.1%)
1000–1999, *n* (%)	34 (11.5%)
2000–3999, *n* (%)	18 (6.1%)
> 4000, *n* (%)	7 (2.4%)
No answer, *n* (%)	222 (73.9%)
Age at eye loss (years), mean ± SD (range)	31.97 ± 23.93 (range, 0–86)
Time since eye loss (years), mean ± SD (range)	30.56 ± 24.67 (range, 0–89)
Reason for eye loss	
Congenital, *n* (%)	16 (5.4%)
Trauma, *n* (%)	151 (51.2%)
Medical: malignant disease, *n* (%)	49 (16.6%)
Medical: no malignant disease, *n* (%)	79 (26.8%)
Operation	
Enucleation, *n* (%)	259 (88.8%)
Evisceration, *n* (%)	22 (3.7%)
None (phthisis/microphthalmos), *n* (%)	25 (8.5%)
Diagnosed depression	
Yes, *n* (%)	20 (6.8%)
No, *n* (%)	275 (93.2%)
Diagnosed anxiety disorder	
Yes, *n* (%)	5 (98.3%)
No, *n* (%)	290 (1.7%)

Reasons for eye loss included accident (51.2%), medical (43.4%), and congenital (5.4%). A percentage of 16.6% had a malignant disease resulting in eye loss. A total of 87.5% of the patients were enucleated, 3.7% eviscerated, and 8.8% had no operation and still retained their blind disfigured globe. Mean age at eye loss was 31.97 ± 23.93 years (range, 0–86 years), and mean time since eye loss was 30.56 ± 24.67 years (range, 0–89 years).

Of the 295 study participants, 20 patients (6.8%) had active pre-diagnosed depression. While 1 of these 20 patients had no current therapy, 4 had only current pharmacological treatment, 9 only current psychotherapy, and 6 combined pharmacological and psychological treatment at the moment. In addition, 5 patients (1.7%) had a pre-diagnosed anxiety disorder, with 4 of them receiving psychotherapy, and the remaining having no treatment at all. All patients with a pre-diagnosed anxiety disorder or depression had currently a stable disease status.

### Depression and anxiety symptoms in prosthetic eye wearers

Mean PHQ-9 score of all 295 patients was 3.01 ± 3.83 and within normal range. Mean PHQ-9 score was 1.28 ± 1.43 in mentally well patients and 8.49 ± 3.91 in patients with depression symptoms with significantly lower scores in mentally well patients. While 224 patients (75.9%) had no depression symptoms (PHQ-9 score 0–4), 71 patients (24.1%) had significant depression symptoms (PHQ-9 score ≥ 5; Table 2). Of these 71 patients, 51 (17.3% of all patients) had mild, 13 (4.4%) moderate, 6 (2.0%) moderately severe, and 1 patient (0.3%) severe depression scores.

**Table 2:** PHQ-9, SF-12, FACE-Q, and VF-14 scores of 295 unilateral anophthalmic patients graded in patients without anxiety and patients with mild, moderate, moderately severe, and severe depression symptoms

	Patients, n (%)	PHQ-9, mean ± SD (range)	SF-12 PCS, mean ± SD (range)	SF-12 MCS, mean ± SD (range)	FACE-Q distress, mean ± SD (range)	FACE-Q social, mean ± SD (range)	VF-14, mean ± SD (range)
All anophthalmic patients (scores 0–27)	295 (100. 0%)	3.01 ± 3.83 (0–21)	47.76 ± 10.0 (17.7–64.8)	52.98 ± 8.81 (15.6–67.1)	8.23 ± 18.91 (0.0–100.0)	80.96 ± 22.23 (0.0–100)	81.46 ± 25.74 (0.0–100.0)
Anophthalmic patients without depression (0–4)	224 (75.9%)	1.28 ± 1.43 (0–4)	49.02 ± 8.92 (17.7–64.8)	55.59 ± 6.11 (31.6–67.1)	5.76 ± 16.61 (0.0–100.0)	84.44 ± 18.75 (38.0–100.0)	83.74 ± 24.18 (0.0–100.0)
Anophthalmic patients with depression (5–27)	71 (24.1%)	8.49 ± 3.91 (5–21)	43.81 ± 12.06 (21.0–61.0)	44.74 ± 10.79 (15.6–65.1)	16.04 ± 23.26 (0.0–70.0)	69.99 ± 28.19 (0.0–100.0)	74.45 ± 29.19 (0.0–100.0)
Mild depression (5–9)	51 (17.3%)	6.39 ± 1.30 (5–9)	45.62 ± 11.83 (21.0–61.0)	48.47 ± 8.96 (24.0–65.1)	13.84 ± 21.01 (0.0–70.0)	71.75 ± 26.33 (0.0–100.0)	74.05 ± 29.91 (0.0–100.0)
Moderate depression (10–14)	13 (4.4%)	12.00 ± 1.63 (10–14)	36.94 ± 9.83 (24.6–57.6)	38.70 ± 7.75 (24.6–49.7)	18.23 ± 26.84 (0.0–70.0)	72.00 ± 31.34 (0.0–100.0)	72.54 ± 31.09 (3.6–100.0)
Moderately severe depression (15–19)	6 (2.0%)	16.67 ± 1.51 (15–18)	45.23 ± 14.79 (25.0–45.2)	30.03 ± 8.73 (15.6–39.4)	32.67 ± 31.40 (0.0–61.0)	54.67 ± 37.35 (8.0–100.0)	80.14 ± 24.7 (32.1–98.2)
Severe depression (20–27)	1 (0.3%)	21.00 ± 0.00 (21–21)	32.22 ± 0.00 (32.2–32.2)	21.09 ± 0.00 (21.1–21.1)	0.00 ± 0.00 (0.0–0.0)	46.00 ± 0.00 (46.0–46.0)	71.43 ± 0.0 (71.4–71.4)

Mean GAD-7 score of all patients was 2.90 ± 4.34, again within the normal range, including 229 (77.6%) patients with minimal anxiety symptoms (GAD-7 score 0–4; Table 3). Mean GAD-7 score of the non-anxious patients was 0.93 ± 1.36 and 9.76 ± 4.15 of the symptomatic patients (GAD score ≥ 5) with significantly higher scores in the symptomatic group. Sixty-six patients had significant anxiety symptoms (GAD-7 score ≥ 5) with 39 patients (13.9%) showing mild, 13 (4.4%) moderate, and 14 (4.7%) severe anxiety symptoms.

**Table 3:** GAD-7, SF-12, FACE-Q, and VF-14 scores of 295 unilateral anophthalmic patients graded in patients without anxiety and patients with mild, moderate, and severe anxiety symptoms

	Patients, n (%)	GAD-7, mean ± SD (range)	SF-12 PCS, mean ± SD (range)	SF-12 MCS, mean ± SD (range)	FACE-Q distress, mean ± SD (range)	FACE-Q social, mean ± SD (range)	VF-14, mean ± SD (range)
All anophthalmic patients	295 (100. 0%)	2.90 ± 4.34 (0–21)	47.76 ± 10.0 (17.7–64.8)	52.98 ± 8.81 (15.6–67.1)	8.23 ± 18.91 (0.0–100.0)	80.96 ± 22.23 (0.0–100)	81.46 ± 25.74 (0.0–100.0)
Anophthalmic patients without anxiety (scores 0–4)	229 (77.6%)	0.93 ± 1.36 (0–4)	48.25 ± 9.66 (17.7–64.8)	55.28 ± 6.49 (31.5–67.1)	5.45 ± 16.18 (0.0–100.0)	84.78 ± 19.39 (0.0–100.0)	82.40 ± 25.18 (0.0–100.0)
Anophthalmic patients with anxiety (scores 5–27)	66 (22.4%)	9.76 ± 4.15 (5–21)	46.09 ± 10.99 (21.6–61.0)	44.97 ± 10.92 (15.6–63.1)	17.88 ± 24.01 (0.0–70.0)	67.73 ± 26.22 (0.0–100.0)	28.19 ± 27.58 (0.0–100.0)
Mild anxiety (scores 5–9)	39 (13.2%)	6.90 ± 1.61 (5–9)	46.94 ± 10.53 (21.6–59.8)	50.00 ± 7.81 (32.5–63.1)	16.51 ± 22.64 (0.0–67.0)	71.31 ± 25.02 (0.0–100.0)	78.37 ± 30.04 (0.0–100.0)
Moderate anxiety (scores 10–14)	13 (4.4%)	11.54 ± 1.05 (10–13)	41.89 ± 11.76 (24.7–58.5)	42.22 ± 7.78 (31.6–57.4)	25.08 ± 28.24 (0.0–70.0)	68.62 ± 24.63 (38.0–100.0)	80.38 ± 21.96 (25.0–100.0)
Severe anxiety (scores 15–21)	14 (4.7%)	16.07 ± 2.59 (14–21)	47.61 ± 11.42 (26.7–61.0)	33.53 ± 11.76 (15.6–55.3)	15.00 ± 24.14 (0.0–61.0)	56.93 ± 29.70 (8.0–100.0)	75.68 ± 26.61 (0.0–100.0)

In total, 198 of 295 (67.1%) had no symptoms, while 31 patients (10.5%) had only depression symptoms, 26 patients (8.8%) had only anxiety symptoms, and 40 patients (13.6%) had both depression and anxiety symptoms. There seems to be a significant underdiagnosing for both depression and anxiety disorders (*p* < 0.001, respectively).

### Associations of explanatory variables with depression and anxiety symptoms

**Fig. 1:**
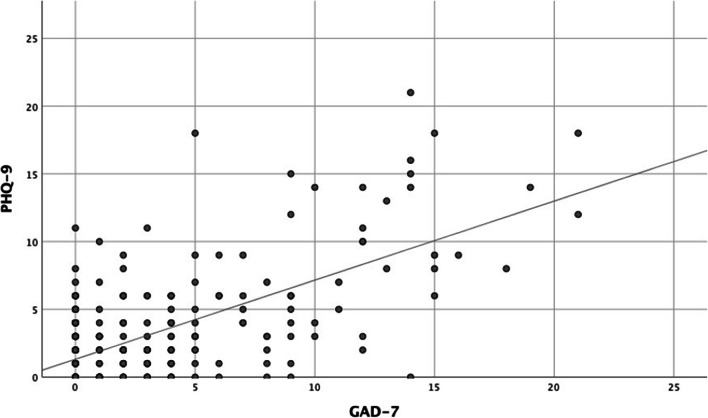
Patients with higher PHQ-9 score also had higher GAD-7 scores

There was a significant positive correlation between PHQ-9 scores and GAD-7 scores (*p* < 0.001; Table 4; Fig. 1). Furthermore, there was a significant association between PHQ-9 scores and both the general MCS and PCS, with higher PHQ-9 scores associated with lower MCS and PCS (*p* < 0.001 and *p* = 0.015, respectively). In addition, higher educational degree and non-traumatic eye loss were associated with higher PHQ-9 scores (*p* = 0.038 and *p* = 0.033, respectively).

**Table 4:** Associations of explanatory variables with PHQ-9 scores of 295 anophthalmic patients

Explanatory variable	Beta coefficient	95% confidence limits	*p* value
GAD-7 score	0.378	0.245 to 0.423	< 0.001
FACE-Q appearance-related distress score	0.041	− 0.009 to 0.026	0.344
FACE-Q social function score	− 0.050	− 0.024 to 0.006	0.262
VF-14 score	− 0.007	− 0.015 to 0.013	0.876
SF-12 PCS	− 0.116	− 0.080 to − 0.009	0.015
SF-12 MCS	− 0.369	− 0.205 to − 0.117	< 0.001
Gender (male [0] vs. female [1])	0.071	− 0.101 to 1.237	0.096
Age	0.055	− 0.010 to 0.035	0.268
Age at eye loss	− 0.012	− 0.017 to 0.013	0.802
Highest educational degree	0.084	0.011 to 0.380	0.038
European (or not)	0.032	− 0.712 to 1.536	0.471
Occupational disability (or not)	0.070	− 0.163 to 2.545	0.084
Reason for eye loss: trauma (or not)	− 0.110	− 1.611 to − 0.070	0.033
Reason for eye loss: tumor (or not)	− 0.018	− 1.092 to 0.716	0.683
Single (or not)	0.014	− 0.907 to 1.132	0.828
Living alone (or not)	− 0.024	− 1.224 to 0.833	0.709
Time since eye loss < 12 months (or not)	− 0.072	− 2.043 to 0.195	0.105

Higher GAD-7 scores were associated with lower appearance-related social function scores (*p* = 0.021) and lower general MCS (*p* < 0.001; Table 5). While there were no associations between gender and the PHQ-9 scores (*p* = 0.096), gender was associated with the GAD-7 scores, with females having significantly higher GAD-7 scores than males (*p* = 0.002).

**Table 5:** Associations of explanatory variables with GAD-7 scores of 295 anophthalmic patients

Explanatory variable	Beta coefficient	95% confidence limits	*p* value
FACE-Q appearance-related distress score	− 0.023	− 0.028 to 0.018	0.056
FACE-Q social function score	− 0.119	− 0.043 to − 0.004	0.021
VF-14 score	0.089	− 0.003 to 0.034	0.111
SF-12 PCS	− 0.076	− 0.080 to 0.014	0.170
SF-12 MCS	− 0.512	− 0.302 to − 0.202	< 0.001
Gender (male [0] vs. female [1])	0.149	0.482 to 2.227	0.002
Age	− 0.110	− 0.058 to 0.001	0.057
Age at eye loss	− 0.051	− 0.029 to 0.011	0.364
Highest educational degree	0.049	− 0.115 to 0.373	0.300
European (or not)	0.040	− 0.892 to 2.085	0.431
Occupational disability (or not)	0.064	− 0.556 to 3.022	0.176
Reason for eye loss: trauma (or not)	− 0.084	− 1.747 to 0.288	0.159
Reason for eye loss: tumor (or not)	0.024	− 0.916 to 1.481	0.643
Single (or not)	− 0.070	− 1.975 to 0.723	0.362
Living alone (or not)	0.117	− 0.261 to 2.454	0.113
Time since eye loss < 12 months (or not)	0.035	− 0.980 to 1.985	0.505

There were no associations between both the PHQ-9 and GAD-7 scores and the explanatory variables of vision-related quality of life, age, age at eye loss, ethnicity, occupational disability, relationship status, time since eye loss, or if patient lived alone (*p* ≥ 0.05, respectively; Tables 4 and 5).

## Discussion

The present study reveals three important findings having significant clinical implications for anophthalmic patients wearing prosthetic eyes:

1. Anxiety and depression disorders seem to be underdiagnosed in the prosthetic eye wearers. Therefore, standardized psychometric screening regarding these depression and anxiety disorders should be implemented in the routine of clinical care.
2. Since the physical condition seems to have a significant influence on depression symptoms, prosthetic eye wearers need not only good ophthalmological and ocularistic care but also good and professional general healthcare.
3. For a successful social and psychological rehabilitation and an interprofessional long-term care of patients wearing prosthetic eyes, an integrated care by a multidisciplinary team including ophthalmic plastic surgeons, ophthalmologists, ocularists, general practitioners, and psychologists is essential and is a high priority to be established in a standardized fashion.

Since the mean duration of prosthesis wear was longer than 30 years, the participants of this study had a lot of experience and knowledge of living with a prosthetic eye and the resulting psychological consequences including depression and anxiety. In addition, they likely had relevant and deep insights into quality of life–affecting factors such as general health status, appearance-related distress, appearance-related social function, and vision-related quality of life. The demographic data of the 295 consecutive enrolled patients was very similar to the data of previous studies and was therefore representative for the ordinary anophthalmic population in Germany quite well [3, 4, 13]. However, limitations of this study include the rather high proportion of patients with enucleation in relation to eviscerations or not operated patients as well as the design as a monocenter study conducted at an ocularistic institute. Patients with severe anxiety and depression might not attend appointments at the ocularists due to their high disorder severity.

Of the 295 study participants, 20 patients (6.8%) had a pre-diagnosed depression, indicating that patients wearing prosthetic eyes have no higher incidence of depression in comparison with the general population [28, 30, 31]. However, the results of our screening with PHQ-9 are in contrast to that and showed that 24.1% of these patients had significant depression symptoms. This suggests that there is a noticeable number of patients that are underdiagnosed. Since 51 of 71 symptomatic patients had only mild symptoms, a psychological or pharmacological treatment is probably not necessary in every case, but of course, these patients should be seen and individually counseled by a psychologist, and if necessary, treatment should be initiated.

A previous study with only 20 patients and without long-term follow-up reported a higher incidence of depression symptoms especially in the first months after enucleation in patients with uveal melanoma [41]. Three months after enucleation due to uveal melanoma, 45% of these patients had mild depression, 25% moderate depression, and 10% severe depression symptoms. In our study with a mean time of more than 30 years since eye loss, 17% of all prosthetic eye wearers had mild, 4% moderate, 2% moderately severe, and 0.3% severe depression scores. These results might indicate that depression symptoms seem to decrease over time. However, the study populations and the methodology in both studies were very different, and the results in this study with a much higher patient number and a long mean time since eye loss showed that time since eye loss had no statistical influence on depression symptoms.

Furthermore, a lower SF-12 PCS was associated with higher depression symptoms, which confirms the results of previous studies in general populations [42]. Therefore, prosthetic eye wearers seem to need not only good ophthalmological and ocularistic care but also professional general healthcare to avoid depression symptoms.

Non-traumatic eye loss was also associated with higher depression symptoms. A reason could be that patients with medical or congenital eye loss have a longer disease history. This could lead to higher depression symptoms [42], but the exact reasons stay unclear like the nature of why patients with higher educational degree had higher depression symptoms. This finding is also in contrast to previous studies in general populations [43].

The significant association between depression and anxiety symptoms in this population is similar to results in general populations [28, 30, 31]. Since prosthetic eye wearers had a higher incidence of depression symptoms, the logical consequence is also the higher incidence of anxiety symptoms in this study. There are not only a noticeable number of patients that are underdiagnosed regarding depression but also a high number of patients with undetected anxiety symptoms, which again confirms the need for professional long-term psychological care. Gender was associated with higher anxiety symptoms similarly to what has been reported for the general population, with females having more anxiety than males [29, 44]. Therefore, special attention should be given to the mental health status of females wearing prosthetic eyes. In addition, there was also a significant association between lower appearance-related social function and anxiety. This is in accordance with the findings of previous studies and suggests that the restoration of facial appearance through good ocularistic care has a significant influence on social interactions and acceptance, resulting in better quality of life and better general mental health [1, 44–46].

In summary, anxiety and depression disorders seem to be underdiagnosed in the prosthetic eye–wearing population. A standardized psychometric screening regarding these depression and anxiety disorders should be implemented in the routine of clinical care. For the successful social and psychological rehabilitation of these patients, long-term, integrated care by a multidisciplinary team including ophthalmic plastic surgeons, ophthalmologists, ocularists, general practitioners, and psychologists is essential.

## Data Availability

The data that support the findings of this study are available on request from the corresponding author. The data are not publicly available due to privacy or ethical restrictions.
